# Ordinal Clinical Outcome Modeling with Temporal Validation to Support Hospital Capacity Planning During Acute Infectious Disease Burden

**DOI:** 10.3390/ijerph23040496

**Published:** 2026-04-14

**Authors:** Tsolmon Sodnomdavaa, Uyanga Gantumur

**Affiliations:** Department of Finance and Economics, Mandakh University, Ulaanbaatar 16061, Mongolia; tsolmon@mandakh.edu.mn

**Keywords:** acute infectious diseases, ordinal machine learning, temporal generalizability, future holdout validation, trustworthy artificial intelligence, clinical decision support systems

## Abstract

**Highlights:**

**Public health relevance—How does this work relate to a public health issue?**

- Acute infectious disease surges place sustained pressure on hospital bed capacity, workforce allocation, and treatment prioritization, requiring reliable early risk stratification tools.
- This study models recovery as an ordinal outcome and evaluates performance under strict temporal validation to reflect real-world public health deployment conditions.

**Public health significance—Why is this work of significance to public health?**

- As a methodological proof-of-concept, the framework preserves the ordered structure of recovery levels and demonstrates that most misclassifications occur between adjacent levels rather than across large ordinal distances.
- Future holdout (temporal) validation provides realistic estimates of model generalizability under evolving epidemiological conditions.

**Public health implications—What are the key implications or messages for practitioners, policy makers and/or researchers in public health?**

- Ordinal modeling frameworks, once validated prospectively with richer clinical data, may contribute to hospital capacity planning and bed management; the current study provides a methodological proof-of-concept.
- The study provides a deployment-oriented, trustworthy machine learning framework integrating calibration, uncertainty estimation, and explanation stability for public health decision support.

**Abstract:**

Acute infectious diseases represent a persistent public health burden that exerts sustained pressure on hospital bed capacity, treatment resources, and the allocation of the healthcare workforce. Strengthening hospital-level preparedness and resource planning requires reliable early-risk stratification tools that remain robust to real-world temporal shifts. However, many existing clinical prediction studies simplify inherently ordered outcomes into binary categories and rely on random data splits, limiting their relevance for real-world health system decision-making. In this study, we developed and evaluated an ordinal machine learning framework using clinical data from 5066 patients hospitalized with acute infectious diseases between 2022 and 2024. Recovery trajectories were modeled as an ordinal outcome, reflecting changes in status between admission and discharge. Models were trained on 2022–2023 data and externally evaluated on a fully isolated 2024 cohort to assess temporal generalizability under realistic deployment conditions. Performance was evaluated using order-aware metrics, including Quadratic Weighted Kappa, Macro-F1, Balanced Accuracy, and ordinal mean absolute error, with explicit analysis of clinically meaningful error structures. Although predictive performance under future holdout validation was modest, misclassifications were predominantly concentrated between adjacent recovery levels, and no clinically critical extreme errors were observed. Model reliability was further assessed through calibration analysis, bootstrap-based uncertainty estimation, and temporal stability of explanatory patterns. Finally, ordinal predictions were translated into structured risk stratification categories aligned with hospital bed management, treatment prioritization, and workforce allocation logic. These findings demonstrate the methodological potential of temporally validated ordinal modeling as a proof-of-concept framework. Given the modest predictive performance and the absence of key clinical variables, the current model should not be regarded as a ready-made clinical decision-support tool, but rather as a foundation for further development with richer data in future research. monitoring prioritization. In practical terms, this framework demonstrates how ordinal predictions could, in principle, be structured for use at admission points. However, given the modest predictive performance observed, further development with richer clinical data is required before deployment.

## 1. Introduction

Acute infectious diseases remain a significant public health burden, periodically generating increased hospital admissions and placing sustained pressure on healthcare systems. Beyond their direct clinical impact, these conditions affect hospital bed turnover, treatment resource allocation, workforce coordination, and overall system resilience. During periods of heightened infectious disease burden, healthcare systems must balance patient-level clinical needs with operational capacity constraints. In this context, early assessment of patients’ treatment trajectories, particularly their anticipated clinical status at discharge, is important not only for individual clinical decision making but also for hospital capacity planning and broader health system preparedness. Nevertheless, developing prognostic models that are applicable in routine clinical settings and remain reliable when applied to future patient populations remains a challenge [1].

To provide a more intuitive understanding, the approach used in this study can be described simply. The model uses routinely collected clinical information at admission to estimate the likely level of patient recovery at discharge. These estimates are expressed as ordered risk categories, which may help clinicians and hospital managers anticipate patient trajectories and plan resources in advance.

Healthcare systems have long encountered structural limitations in responding to sudden increases in patient demand. Prior evidence suggests that commonly used indicators, such as annual hospital bed statistics, may not adequately reflect actual surge capacity under real-world conditions [2]. In addition, earlier work has highlighted the importance of coordinated strategies at both the facility and community levels, including staffing flexibility and resource reallocation, to manage patient surges more effectively [3].

In recent years, the expansion of clinical databases, particularly electronic health record systems, has facilitated the application of machine learning methods for clinical prediction, risk stratification, and disease course modeling [4]. However, systematic reviews have identified substantial variation in study design and methodological rigor, with validation approaches often not aligned with real-world deployment settings. These findings indicate that strong predictive performance alone does not necessarily translate into practical clinical usefulness [5]. In particular, models evaluated using random data splits may perform well under controlled conditions but show reduced reliability when applied to future data due to temporal changes in patient characteristics and clinical practice [6].

Infectious disease outbreaks also exhibit marked variation across both space and time. Evidence from recent studies shows that disease clusters can emerge unevenly across regions and periods, placing disproportionate pressure on specific healthcare facilities [7]. Such variability complicates planning efforts and underscores the need for approaches that anticipate demand changes rather than react to them after they occur. In addition, weighted population density, which accounts for spatial distribution rather than simply averaging, provides a more refined representation of transmission risk and helps explain why some areas experience greater pressure on healthcare systems than others.

Many clinical outcomes used to describe treatment response are not binary but follow an ordinal pattern. A patient’s condition may improve gradually, moving across several levels rather than shifting between two states. Despite this, it remains common to simplify such outcomes into binary or nominal categories. This simplification can obscure clinically relevant differences and may lead to misleading interpretations of prediction errors, particularly when small and large deviations are treated as equivalent [8]. For this reason, maintaining the ordinal nature of outcomes, together with appropriate evaluation measures, has been increasingly recognized as a more suitable approach in clinical modeling [9].

In addition to predictive performance, models intended for clinical use should provide information that supports interpretation and decision-making. This includes an assessment of uncertainty and an explanation of the factors influencing predictions. From a practical perspective, uncertainty quantification helps to manage decision risk, while interpretable outputs can facilitate clinical acceptance. At the same time, existing research has pointed out that explanations derived from complex models may not always be stable or reliable, suggesting that interpretability should be considered alongside robustness and consistency [10,11]. Broader discussions in the literature have also emphasized that explainability in healthcare involves not only technical aspects but also considerations related to clinical practice, ethics, and accountability [12].

Although predictive modeling has advanced considerably, its integration into routine clinical and operational decision-making remains limited. In many cases, risk estimates are available, but their translation into concrete actions for capacity planning, resource allocation, or workforce management is less well-defined. Bridging this gap between prediction and application is therefore an important direction for future research.

During periods of system stress, these challenges may become more pronounced, particularly when demand for critical care exceeds available resources. In such situations, structured approaches to prioritization and allocation have been proposed to support decision-making under resource constraints [13].

Against this background, the present study examines recovery outcomes as an ordinal construct using clinical data from patients hospitalized with acute infectious diseases between 2022 and 2024. A key aspect of the study design is the use of temporally separated data, where models trained on earlier observations are evaluated on a later cohort. This approach allows for a more realistic assessment of performance under changing conditions. In addition, the study considers not only predictive accuracy but also the stability of model explanations and the role of uncertainty. Finally, the results are interpreted in terms of clinical risk stratification and their potential relevance for operational planning. In this way, the study seeks to contribute to the development of modeling approaches that are both analytically sound and practically applicable in healthcare settings.

For clarity, key abbreviations used in this study include ML (machine learning), QWK (Quadratic Weighted Kappa), SHAP (Shapley Additive Explanations), and EHR (electronic health records).

## 2. Literature Review

### 2.1. Clinical Prediction and Data-Driven Modeling

In recent years, the expansion of electronic health record systems and clinical data infrastructures has positioned clinical prediction modeling, defined as the estimation of future patient outcomes and risks, as a central focus of data-driven research. In this context, both regression-based approaches and machine learning methods have been increasingly adopted in clinical settings for prognosis and risk assessment [4,14]. However, the value of clinical prediction research is not determined solely by high predictive performance. There is a growing emphasis on transparent reporting across all stages of the modeling process, from development to validation, together with rigorous evaluation of methodological quality, particularly for models intended for real-world clinical use [1].

A foundational framework for standardizing the reporting of prediction model studies is provided by the TRIPOD guideline, which outlines comprehensive and verifiable reporting requirements covering model objectives, data sources, predictor selection and preprocessing, handling of missing data, validation strategies, and performance reporting [15]. In parallel, there has been increasing recognition of the need to systematically assess the risk of bias and the applicability of prediction models in research. The PROBAST tool addresses this need by defining a structured framework that clarifies which methodological aspects should be evaluated, supported by detailed explanation and guidance [16].

As machine learning and artificial intelligence methods have grown in adoption, these foundational frameworks have been expanded and updated. The TRIPOD + AI statement extends reporting guidance to accommodate both regression-based and machine-learning predictive models, placing greater emphasis on information specific to AI systems, including data-flow transparency, clarity in model development and validation procedures, and an explicit description of intended use and limitations [17]. Similarly, PROBAST + AI provides an updated set of criteria tailored to prediction models developed with AI or machine learning, enabling a more granular assessment of risk of bias and applicability in this evolving methodological landscape [18].

In clinical practice, the transition from research to real-world use cannot be achieved solely through statistical performance. Factors such as data quality, alignment with clinical workflows, practical barriers to deployment, and ongoing monitoring play a decisive role in determining clinical impact [19]. In response to these challenges, the CONSORT-AI and SPIRIT-AI extensions were developed to improve transparency in the reporting of clinical trials and study protocols involving artificial intelligence, with particular emphasis on reproducibility, implementation context, and intended use [20,21]. In addition, efforts to standardize how model information is communicated to clinical end users, such as the use of model fact labels, have been shown to provide a practical means of clarifying model purpose, conditions of use, and key limitations, thereby supporting more informed and responsible adoption in clinical settings [22].

### 2.2. Temporal Generalizability and Validation

The generalizability of clinical prediction models extends beyond achieving high performance within a single dataset to encompass reliable operation under new conditions and across future time periods [23,24]. A key approach to realistically evaluating this property is temporal split validation, in which models are trained on earlier data and evaluated on later data. By explicitly accounting for temporal shifts, this strategy enables a more rigorous assessment of model performance under conditions that better reflect real-world clinical deployment [25,26].

Recent studies have cautioned that prediction models may exhibit illusory generalizability, particularly when validation designs are weak or insufficiently aligned with intended use, leading to substantial performance degradation in new clinical environments [6]. In addition, common misconceptions regarding external validation have been clearly articulated, including misclassifying internal validation as external validation and failing to adequately characterize differences between development and validation settings, both of which may result in misleading conclusions [27]. These considerations underscore the importance of clearly distinguishing between validation types, such as temporal, geographical, and domain-based validation, and reporting them directly in relation to the study objectives [17,24].

Beyond purely temporal considerations, infectious disease dynamics are also shaped by spatial heterogeneity and population structure. Urban density and its spatial configuration influence contact patterns and transmission dynamics, suggesting that more refined representations of population distribution are relevant for understanding variation in disease spread [28].

Systematic reviews have further identified recurrent methodological shortcomings in machine-learning-based prediction model studies, including inadequate validation procedures, poorly defined study designs, and incomplete reporting practices. Such limitations pose substantive barriers to the reliable assessment of model generalizability [5]. Consequently, there is growing consensus that clinical prediction models should be developed and evaluated within a coherent methodological framework that explicitly links model development with temporal and external validation, thereby supporting more credible and clinically relevant conclusions [17,25].

### 2.3. Ordinal Clinical Outcomes and Modeling

Many clinical outcomes, including patient severity levels and trajectories of improvement or deterioration, are inherently ordered and therefore represent ordinal outcomes. Forcing such data into binary or nominal multiclass formulations risks discarding essential ordinal information, which is why modeling ordinal outcomes in ways that preserve their natural structure is widely regarded as more appropriate [8]. Proportional odds models are commonly used for the analysis of ordinal outcomes; however, recent clinical trial practice has emphasized that the proportional odds assumption may not hold consistently in real-world data. This has led to increased attention to formally testing this assumption and conducting sensitivity analyses when it is violated [29,30].

A scoping review examining the statistical analysis of ordinal outcomes in clinical trials has systematically documented variation in reporting practices, methodological choices, and practical challenges associated with ordinal analyses [31]. In addition, the development of standardized ordinal severity scales for retrospective COVID-19 studies using electronic health record data underscores the importance of defining ordinal outcomes that are both clinically meaningful and aligned with real-world data structures [32].

From a machine learning perspective, training models with ordinal target variables in conventional multiclass settings can lead to a loss of ordinal structure. This underscores the need for order-aware evaluation strategies and metrics that reflect clinically meaningful error structures [33]. Among these, weighted kappa measures, particularly Quadratic Weighted Kappa, enable differential weighting of near and distant misclassifications and are therefore considered more suitable for the clinical interpretation of ordinal predictions [9].

Furthermore, heterogeneity in clinical outcomes may also arise from differences in demographic structure and disease characteristics. Evidence from COVID-19 studies indicates substantial variation in age-specific mortality and immunity patterns, highlighting the importance of accounting for such differences when modeling clinical trajectories [34]. Such heterogeneity may also reflect differences across viral variants, which have been shown to exhibit distinct age profiles and clinical characteristics over time, further influencing observed patterns of disease severity and recovery.

Accordingly, studies involving ordinal outcomes should incorporate modeling approaches consistent with ordinal logic, explicitly assess the proportional odds assumption when applicable, and report performance using order-sensitive metrics to produce results that are both clinically interpretable and reusable across settings [8,30,33].

### 2.4. Trustworthy Machine Learning: Reliability, Uncertainty, and Explanation Stability

For machine learning models to be used effectively for clinical decision support, accuracy alone is insufficient. Consideration must be given jointly to reliability, control of uncertainty, and explainability-related risks [12,35]. First, calibration represents a central pillar of reliability in prediction models. When predicted probabilities do not align with observed outcome frequencies, systematic errors may arise in clinical decision-making, even in models with apparently good discrimination [36].

Second, uncertainty quantification enables the distinction between situations in which predictions can be acted upon with confidence and those in which caution is warranted. A comprehensive review of uncertainty quantification methods in deep learning has highlighted their techniques, applications, and challenges, emphasizing the growing need to report uncertainty in high-risk domains such as clinical care [10]. In this context, Bootstrap-based confidence intervals are employed in this study to quantify uncertainty in performance estimates under shifts in temporal distributions [37].

In addition, temporal and biological variability may contribute to uncertainty in predictions. Seasonal changes in human immune function, as demonstrated by large-scale gene expression studies, indicate that susceptibility to disease may vary over time, further complicating predictive modeling [38].

Third, although post hoc explanation methods are widely used in clinical settings, their robustness and susceptibility to misleading effects require careful consideration. Foundational work on SHAP and subsequent extensions demonstrating the transition from local to global explanations in tree-based models have supported the practical adoption of explainability in applied settings [39,40]. However, empirical evidence showing that post hoc explanations may be insufficient for detecting unknown spurious correlations has raised strong concerns regarding the reliability of explanations themselves, underscoring the need to evaluate explanation robustness as a distinct methodological dimension [11]. Moreover, studies focusing on explainable machine learning in deployment contexts have emphasized that explanation methods interact with organizational workflows, monitoring practices, and governance mechanisms, introducing additional challenges when models are integrated into real-world clinical environments [41].

Accordingly, within the framework of trustworthy machine learning, calibration, uncertainty, and explanation-related risks should be considered jointly rather than in isolation. Addressing these dimensions together is increasingly recognized as a foundational requirement for transforming clinical prediction models into reliable decision-support systems that can inform real-world clinical practice [10,35,36].

### 2.5. Decision Support, Translation, and Research Questions

The practical value of clinical prediction models is determined not only by performance metrics but also by the extent to which predictions can be translated into actionable decisions within clinical workflows [4,19]. Accordingly, standardizing how model objectives, conditions of use, limitations, explanations, and monitoring requirements are communicated to clinical end users is critical to the quality and sustainability of real-world deployment [22]. In parallel, evaluation frameworks that integrate transparent reporting, rigorous validation, and external validation logic have been shown to enhance the likelihood that clinical prediction models can be meaningfully translated from research settings into routine practice [17].

Ordinal outcomes offer particular advantages for decision support translation. By expressing risk along ordered levels, ordinal predictions facilitate more direct alignment with clinical interpretation, resource planning, and triage decisions [31,32]. However, such translation is only clinically meaningful when supported by robust temporal generalizability, rigorous validation design, and trustworthy modeling practices, including calibration and explicit uncertainty reporting [26,36,37].

Synthesizing the above considerations, it becomes evident that clinical prediction modeling requires the systematic integration of: (i) temporal generalizability, (ii) modeling and evaluation strategies aligned with ordinal outcomes, (iii) joint consideration of calibration, uncertainty, and explainability risks, and (iv) application-oriented reporting and documentation frameworks, including TRIPOD and TRIPOD + AI, as well as PROBAST and PROBAST + AI [1,15,16,18].

Accordingly, this study aims to model ordinal clinical outcomes using a rigorous time-based design, to assess model reliability and the stability of explanatory structures, and to empirically examine how ordinal predictions can be translated into risk stratification and resource planning. These objectives are formalized through the following research questions:

RQ1. To what extent do models for predicting ordinal clinical outcomes preserve their performance when evaluated on temporally separated holdout data?

RQ2. How effective are ordinal modeling approaches and rank-aware evaluation metrics (e.g., weighted kappa) in constraining clinically distant misclassifications?

RQ3. How do model calibration and uncertainty estimates (e.g., bootstrap-based confidence intervals) behave under temporal distribution shifts, and what implications do they have for decision-support use?

RQ4. Which deployment-ready forms of ordinal prediction-based risk stratification are most suitable for alignment with clinical decision-making and resource planning logic?

## 3. Methods

In this study, improvement in the clinical course of acute infectious diseases is defined as an ordinal outcome and modeled within a deployment-oriented machine learning pipeline designed to support reliable generalization to future data and direct applicability in real-world clinical decision support systems. The methodological framework was developed to ensure that model outputs remain clinically meaningful, temporally robust, and operationally actionable. The core methodological principles guiding this pipeline were threefold. First, the design aimed to closely approximate real-world clinical use conditions, rather than idealized experimental settings. Second, the entire workflow was structured to systematically prevent data leakage across model development, validation, and evaluation stages. Third, model assessment extended beyond predictive performance alone, incorporating reliability and interpretability considerations to support trustworthy clinical use.

### 3.1. Data Source and Time-Based Study Design

This study adopted a retrospective cohort design based on clinical records of 5066 patients hospitalized at the Third Department of the National Cancer Center between 2022 and 2024. All data were extracted from the hospital’s primary clinical information system, and patient diagnoses were classified using standardized coding according to the International Classification of Diseases, 10th Revision (ICD-10). Patients were grouped by etiology into viral, bacterial, and other/unspecified infection categories. All stages of the study complied with relevant ethical approval requirements, and only fully anonymized data were used. Any information that could enable direct or indirect patient identification was removed before analysis. 

To evaluate model generalizability under real-world use conditions, random training–testing splits were deliberately avoided. Instead, data from 2022–2023 were used for model development, while data from 2024 were reserved as a fully isolated future holdout test set (see Table A3 in Appendix A for the detailed dataset split used in temporal validation). The holdout dataset was not used at any stage of model training, hyperparameter tuning, or model selection. This strict time-based design was intended to emulate real deployment scenarios and prevent systematic information leakage. All stages of data preprocessing, outcome construction, model training, and evaluation were implemented within an end-to-end machine learning pipeline.

### 3.2. Definition of the Ordinal Outcome and Input Features

**Figure A1:**
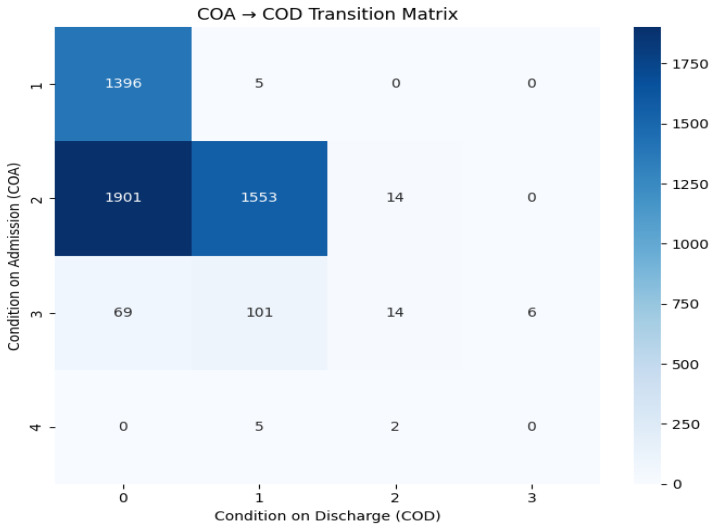
COA → COD transition matrix (heatmap). Heatmap representation of transitions from condition on admission (COA) to condition on discharge (COD). The matrix highlights the concentration of cases in adjacent transitions, supporting the ordinal structure of the outcome variable.

The primary outcome of interest was the level of clinical improvement, defined as an ordinal variable based on the difference between the patient’s condition at admission (Condition on Admission, COA) and condition at discharge (Condition on Discharge, COD). Both COA and COD are recorded on a 4-point ordinal scale (1 = mild, 2 = moderate, 3 = severe, 4 = critical) as documented in the hospital’s clinical information system at the time of each assessment. The recovery level was computed as (COD minus COA) and discretized into four ordinal categories: level 0 (no improvement or deterioration: difference ≤ 0), level 1 (mild improvement: difference = 1), level 2 (moderate improvement: difference = 2), and level 3 (substantial improvement: difference ≥ 3) (see Figure A1 in Appendix A for the transition matrix representation of COA → COD).

Input features were restricted to information that is objectively available at the time of hospital admission. These included demographic characteristics (age and sex), clinical status at admission (COA), etiological classification of infection, and temporal attributes (month and season of admission). Variables with a high proportion of missing values or limited clinical relevance were excluded during the modeling stage to preserve robustness and interpretability.

### 3.3. Ordinal Machine Learning Modeling and Evaluation

Because the clinical improvement outcome exhibits a natural ordering across categories, the prediction task was formulated as an ordinal outcome problem rather than a conventional multiclass classification task. As a statistical baseline, an ordered logistic regression model was employed to establish a reference level of interpretability and to examine the direction and relative influence of covariates.

To improve accessibility for non-technical readers, the modeling approach can be interpreted as a structured process in which patient information available at admission is used to estimate the likely level of recovery at discharge, and these estimates are evaluated on future data to reflect real-world deployment conditions.

In parallel, a comparative analysis was conducted using machine learning models commonly applied to tabular clinical data, including Gradient Boosting, LightGBM, CatBoost, and Random Forest, each configured to account for the ordinal structure of the outcome when applicable. To address pronounced class imbalance across outcome levels, class-weighted loss functions and sample weighting strategies were incorporated during model training.

Model performance was evaluated solely on the fully isolated 2024 future holdout dataset to assess generalizability under realistic deployment conditions. Evaluation metrics were selected to reflect the ordinal nature of the task and included Quadratic Weighted Kappa (QWK), Macro-F1 score, Balanced Accuracy, and Ordinal Mean Absolute Error (MAE). In addition, transition patterns and error distributions across outcome levels were examined in detail, with particular attention to misclassifications involving clinically consequential severity levels. To ensure alignment between methodological rigor and real-world deployment constraints, the following end-to-end machine learning pipeline was implemented in a Python 3.10 environment (Python Software Foundation, Wilmington, DE, USA) using Google Colab (Google LLC, Mountain View, CA, USA) and standard machine learning libraries, including scikit-learn (version 1.3.2), LightGBM (Microsoft Corporation, Redmond, WA, USA; version 4.1.0), CatBoost (Yandex LLC, Moscow, Russia; version 1.2.2), and SHAP (version 0.44.1): Clinical records were chronologically ordered based on the admission date to preserve the temporal structure of the data.

- Rule-based encoding was applied exclusively to admission-time variables to reflect information availability at the point of clinical decision-making.
- A strict temporal split was enforced, with data from 2022–2023 used for model development and data from 2024 reserved as a fully isolated future holdout set.
- An ordinal outcome variable was constructed based on the difference between COA and COD, capturing the ordered nature of clinical improvement.
- Tree-based models were trained and compared using class-weighted configurations to account for class imbalance.
- Model selection and hyperparameter tuning were performed solely on the training data.
- A single, final evaluation was conducted on the future holdout dataset to assess deployment-level performance.
- Model stability, uncertainty, and robustness were evaluated using SHAP-based analyses and bootstrap resampling.

### 3.4. Reliability, Uncertainty, and Reproducibility

Model reliability was assessed as a system-level property rather than limited to post hoc interpretability. The stability of feature influence was examined using SHAP-based bootstrap resampling, and the consistency of feature importance rankings between the training and holdout periods was quantified using Spearman’s rank correlation. Predictive uncertainty was evaluated by constructing bootstrap-based 95% confidence intervals for each performance metric, thereby capturing sampling variability. In addition, probabilistic reliability was assessed through calibration curves computed on the future holdout dataset, enabling examination of the alignment between predicted probabilities and observed outcome frequencies under deployment-like conditions. All experiments were conducted in the same Python 3.10 environment and software setup described in Section 3.3. To ensure reproducibility, fixed random seeds were applied to all stochastic procedures. Although the underlying clinical data cannot be publicly shared due to privacy constraints, the methodological framework, feature encoding logic, and evaluation protocols described in this study are fully reproducible on structurally comparable datasets.

## 4. Results

### 4.1. Temporal Generalization Performance

This subsection evaluates the ability of ordinal prediction models to generalize to future data when predicting recovery levels derived from changes in patients’ clinical condition during hospitalization for acute infectious diseases. The recovery outcome was defined solely based on the ordinal difference between condition on admission (COA) and condition on discharge (COD), ensuring that only information available at admission was used and avoiding leakage from discharge-time variables (e.g., length of stay or final diagnosis). This design choice reflects a deployment-oriented framework aligned with real-world clinical decision-support applications.

Model evaluation followed a strict time-based design without using random data splits. Data from 2022–2023 were used for model training, while data from 2024 were reserved as a fully isolated future holdout test set. This approach enables a realistic and deployment-consistent assessment of model performance under temporal shifts, including changes in seasonality, disease composition, and evolving clinical practice, thereby reducing the risk of overly optimistic performance estimates commonly observed under random splitting strategies.

The distribution of recovery levels in the 2024 holdout dataset (*n* = 1612) was highly imbalanced (see Table A1 in Appendix A for detailed distribution). Specifically, recovery level 0 (no or minimal improvement) accounted for 0.6% of cases, level 1 for 35.3%, level 2 for 62.9%, and level 3 or higher for 1.2%. Such a skewed outcome distribution reflects real-world clinical conditions and provides a stringent test of temporal generalization, rather than inflating performance through artificially balanced evaluation settings. The ordinal performance of the baseline statistical model (Multinomial Logistic Regression) and machine learning models suitable for tabular data is summarized in Table 1.

As shown in Table 1, the overall performance of all models was modest, which is directly attributable to the strict future holdout evaluation design and the pronounced imbalance in the outcome distribution. Unlike overly optimistic estimates derived from random splits, these results more accurately reflect real-world deployment performance. Nevertheless, model strengths varied across evaluation metrics. The Multinomial Logistic Regression model achieved the highest values for Quadratic Weighted Kappa and Balanced Accuracy, indicating relatively strong preservation of ordinal agreement and class-wise discrimination. In contrast, the LightGBM model yielded the lowest Ordinal Mean Absolute Error (0.411), suggesting improved performance in minimizing the average magnitude of ordinal misclassification. The Gradient Boosting model demonstrated the highest Macro-F1 score, reflecting a more balanced trade-off across recovery levels.

To further examine model generalizability, performance was stratified by disease etiology (viral, bacterial, or other/unspecified) (see Table A2 in Appendix A for full etiology distribution). The corresponding results are summarized in Table 2.

As shown in Table 2, the viral subgroup exhibited relatively lower values of Quadratic Weighted Kappa and Macro-F1, likely reflecting greater heterogeneity in disease progression and outcome variability. In contrast, the bacterial subgroup showed higher QWK values, indicating greater stability in ordinal agreement between predicted and observed recovery levels. For the “other/unspecified” group, Balanced Accuracy appeared higher; however, given the relatively small sample size in this subgroup, these results should be interpreted with caution.

Below, we provide an in-depth analysis of ordinal performance and clinically relevant errors.

A key characteristic of ordinal prediction problems is that not all misclassifications carry the same clinical risk. Accordingly, model performance was not assessed solely through aggregated metrics but was examined in greater detail by analyzing error structure and clinically meaningful misclassification patterns.

**Figure 1:**
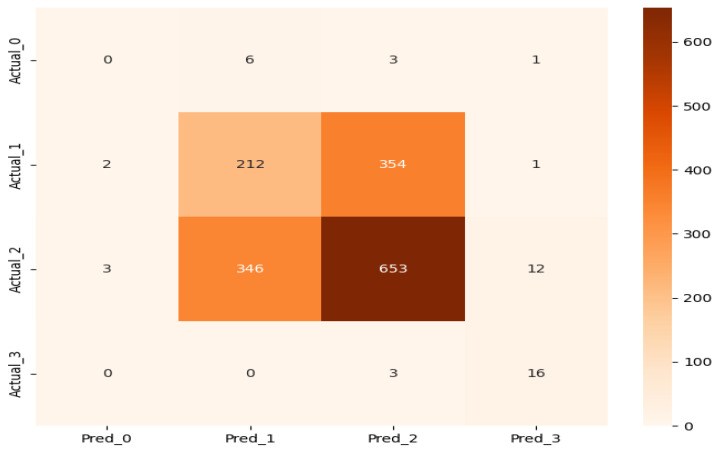
Confusion matrix of ordinal recovery prediction (2024 holdout). Most prediction errors are concentrated between adjacent recovery levels, particularly between levels 1 and 2, indicating preservation of ordinal structure. Importantly, extreme misclassifications (e.g., level 3 predicted as level 0) are extremely rare (only one case observed).

The confusion matrix presented in Figure 1 demonstrates that prediction errors are predominantly confined to adjacent recovery levels, confirming that the model preserves clinically meaningful ordering.

**Figure 2:**
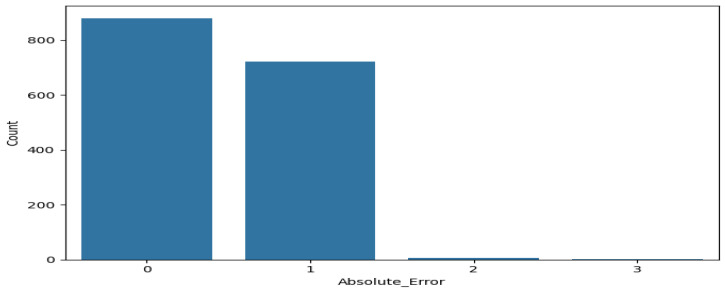
Distribution of absolute ordinal prediction errors |ŷ − y|. The majority of predictions fall within exact or adjacent recovery levels, indicating clinically acceptable error patterns.

To further quantify the clinical relevance of prediction errors, the absolute deviation between predicted and observed recovery levels (|ŷ − y|) was analyzed (Figure 2). Exact agreement (|ŷ − y| = 0) was observed in 54.6% of cases, while 44.6% of predictions deviated by one ordinal level. Errors of two levels accounted for only 0.8%, and three-level misclassifications were extremely rare, with only 1 case observed (Actual = 0, Predicted = 3), representing less than 0.1% of the total holdout set.

As shown in Table 3, no clinically severe misclassifications from level 3 to level 0 or level 1 were observed. A single three-level misclassification (Actual = 0, Predicted = 3; n = 1) was identified, representing less than 0.1% of the total holdout set. Only a small number of level 3-to-level 2 misclassifications were identified (n = 3), corresponding to 0.2% of the 2024 dataset. These errors result from confusion between adjacent recovery levels and therefore pose a relatively moderate clinical risk. Nevertheless, given the limited sample size of patients in the true level 3 category (n = 19), these proportions should be interpreted cautiously.

Overall, these findings indicate that ordinal machine learning models predominantly concentrate prediction errors within clinically adjacent states while systematically avoiding extreme misclassifications associated with high clinical risk. Such an error profile is a positive methodological finding; however, safe integration into real-world clinical decision-support systems requires substantially higher predictive performance than demonstrated here, as well as prospective clinical validation.

### 4.2. Ordinal vs. Binary Modeling Comparison

To further examine the implications of modeling choices, an additional analysis was conducted by reformulating the outcome variable into a binary classification task, distinguishing between “improved” (recovery level ≥ 1) and “not improved” (recovery level = 0). This transformation reflects a simplified clinical decision framework commonly used in practice.

The binary model was trained and evaluated using the same temporal validation design as the ordinal model, with data from 2022–2023 used for training and the 2024 dataset reserved as an out-of-sample holdout set. Model performance was assessed using the Area Under the Receiver Operating Characteristic Curve (AUC), while the ordinal model was evaluated using the Quadratic Weighted Kappa (QWK), which explicitly accounts for the ordered structure of recovery levels and penalizes larger misclassifications more heavily than adjacent errors.

As shown in Table 4, the binary model achieved an AUC of 0.512, which is only marginally above random classification. This suggests that the simplified binary outcome does not capture sufficient discriminative information under temporal validation. In contrast, the ordinal model, while also exhibiting modest QWK values (0.113), preserves the ordered structure of recovery outcomes and enables differentiation between clinically adjacent states. Both models demonstrated limited discriminative ability under strict temporal validation, attributable to the absence of key clinical variables, such as vital signs and laboratory results. These results should be interpreted as a methodological proof-of-concept rather than evidence of clinical readiness.

Importantly, the binary formulation collapses multiple clinically distinct recovery trajectories into a single category, thereby discarding meaningful variation in patient progression. This loss of granularity limits its practical utility, particularly in scenarios where distinguishing between moderate and substantial improvement is critical for resource allocation and clinical decision-making.

This further demonstrates that binary simplification obscures clinically relevant distinctions between recovery levels and reduces interpretability under real-world conditions.

Furthermore, the near-random performance of the binary model indicates that the underlying data structure does not support a simple dichotomous separation between improved and non-improved patients. In contrast, the ordinal framework preserves the graded nature of recovery and aligns more closely with the clinical reality of disease progression.

Taken together, these results provide empirical support for ordinal modeling, particularly in temporal validation settings, where preserving outcome structure is essential for maintaining clinical interpretability and decision relevance.

Overall, these findings demonstrate that although binary classification may offer conceptual simplicity, it is insufficient to capture clinically meaningful recovery dynamics in this context. The ordinal modeling approach provides a more informative and practically relevant representation of patient outcomes, supporting its use in hospital capacity planning and decision-support systems.

### 4.3. Model Reliability and Interpretability

In clinical decision support settings, the use of ordinal prediction models requires consideration beyond raw predictive performance. In particular, the stability of the decision logic, the reliability of predictions, and the extent to which model outputs can be meaningfully interpreted in a clinical context are critical criteria. Accordingly, this subsection evaluates model reliability across three complementary dimensions: (i) stability of feature influence, (ii) temporal stability of the explanatory structure, and (iii) predictive uncertainty.

**Figure A2:**
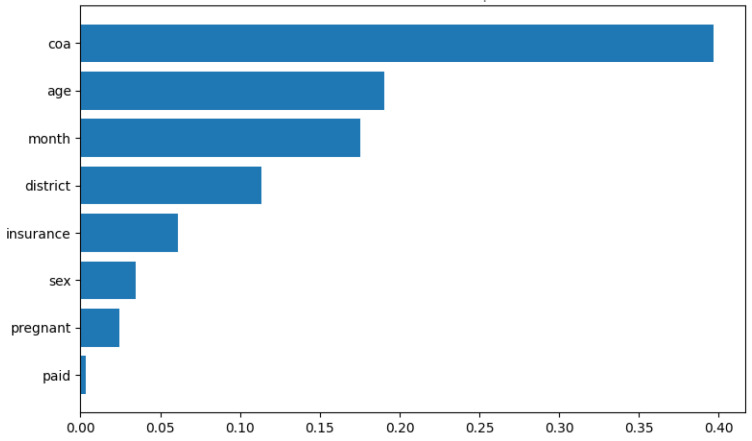
Random Forest feature importance. Feature importance derived from the Random Forest model. The results confirm that condition on admission (COA) is the dominant predictor, followed by age and seasonal factors.

**Figure A3:**
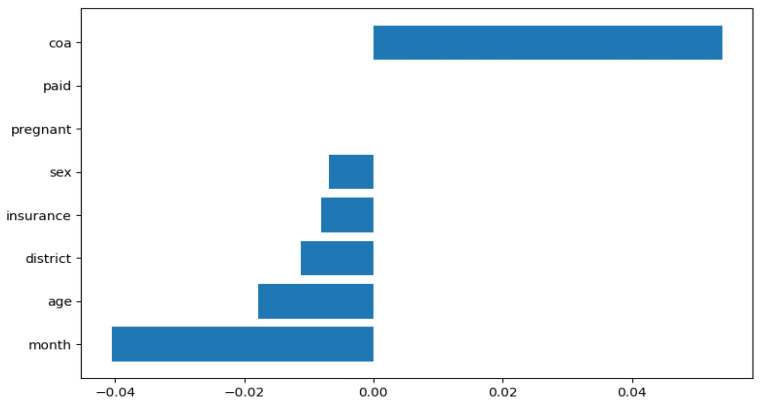
Permutation feature importance. Permutation-based feature importance reflects the change in model performance when individual features are randomly shuffled. Note that some features identified as important by SHAP analysis (notably month and age) show negative permutation importance values, indicating that shuffling these features does not decrease performance. This discrepancy may reflect collinearity among features or instability in the model under permutation and warrants further investigation. The permutation importance results should therefore be interpreted with caution and not assumed to be consistent with the SHAP-based findings.

**Figure A4:**
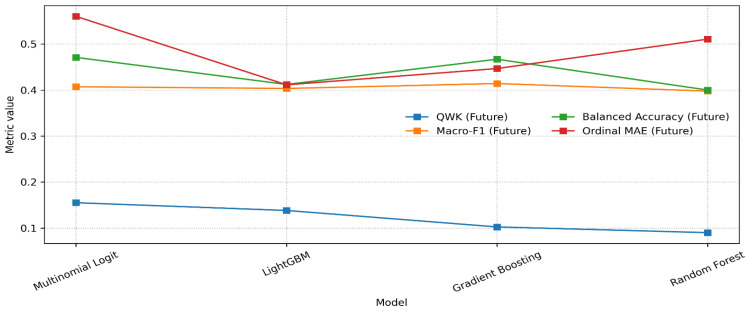
Comparison of ordinal predictive performance across models on the 2024 holdout dataset. Note: A grouped bar chart should replace the current line chart, as the *x*-axis represents discrete, independent machine learning algorithms (LightGBM, CatBoost, Gradient Boosting, Random Forest, Multinomial Logit) rather than a continuous or sequential variable. Line charts imply sequential relationships that do not apply here. Comparison of ordinal predictive performance across models on the 2024 dataset, illustrating differences across QWK, Macro-F1, Balanced Accuracy, and Ordinal MAE.

To assess the stability of feature effects, a bootstrap resampling strategy was applied to the training dataset, and SHAP-based summaries were computed for each resample. Specifically, the mean absolute SHAP value (Mean |SHAP|), average rank position (Mean Rank), and rank variability (Rank SD) were calculated for each input feature. These metrics jointly characterize not only the magnitude of feature influence but also the consistency of their relative importance across resampled training sets. This approach allows for the simultaneous evaluation of both the interpretability and robustness of model explanations under data perturbation. The results are presented in Table 5 (see Figure A2, Figure A3 and Figure A4 in Appendix B for complementary feature importance analyses and model comparison results).

Importantly, SHAP values quantify the contribution of each feature to model predictions and do not represent statistical significance measures. Therefore, they are not directly associated with *p*-values or inferential hypothesis testing frameworks. This distinction reflects the difference between predictive modeling and traditional statistical inference.

As shown in Table 5, the Condition on Admission remained the most influential feature across all bootstrap samples (Mean Rank = 1.00, Rank SD = 0.00), indicating a consistently dominant and stable contribution to model decision-making. Seasonal effects (Month of Admission), Patient Age, District of Residence, Sex, and Insurance Status also consistently ranked among the top 10 features, with relatively low rank variability, suggesting robust and stable influence patterns. In contrast,

Surgical Intervention (at admission) had a Mean |SHAP| of approximately zero and the highest Mean Rank (10.28) among all features, indicating a consistently weak and marginal contribution under admission-time prediction conditions. As the model was evaluated across bootstrap resamples with replacement, this feature occasionally fell below the top-10 threshold when other features exhibited higher variance in ranking. Its Top-10 frequency (45.0%) reflects this instability.

For comparison, conventional statistical models such as logistic regression rely on *p*-values to assess inferential significance, whereas machine learning models emphasize predictive contribution and generalization performance. In this study, SHAP-based analysis is therefore used to evaluate feature importance within a predictive framework rather than to conduct statistical inference.

To further assess the temporal stability of the explanatory structure, SHAP-based feature influence patterns computed on the 2022–2023 training data were compared with those derived from the 2024 future holdout dataset. It should be noted that permutation-based feature importance (Figure A3) yielded divergent results for certain features (notably month and age), with negative permutation importance values observed (see also Figure A2 in Appendix B for Random Forest-based feature importance for comparison). This divergence indicates potential collinearity or model instability under permutation and is acknowledged as a limitation of the current analysis.

**Figure 3:**
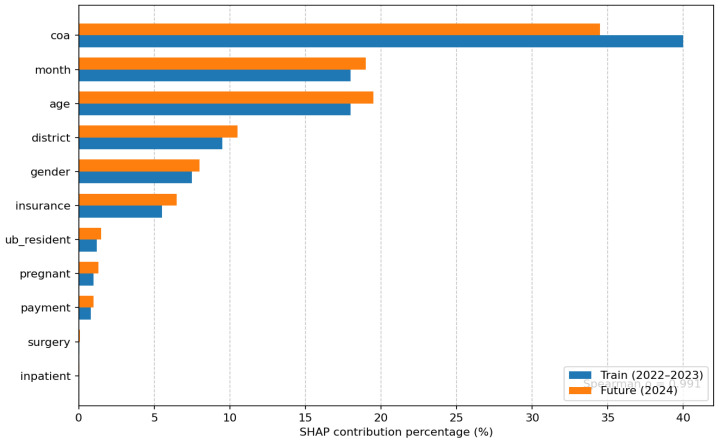
Comparison of SHAP-based feature importance between the training (2022–2023) and future holdout (2024) datasets. The relative importance and ranking of key predictors remain highly consistent across time (Spearman ρ = 0.991), indicating strong temporal stability of model interpretation.

As illustrated in Figure 3, both the direction and relative magnitude of feature influence were largely preserved between the training period and the future holdout dataset. In particular, the positions of the key predictors, including condition on admission (COA), month, and age, remained highly consistent across the two periods. The resulting Spearman rank correlation coefficient of SHAP importance rankings was ρ = 0.991, indicating a very high degree of temporal stability in the model’s explanatory logic despite the time shift. This level of agreement suggests that the model captures persistent structural relationships rather than time-specific artifacts (see also Figure A4 in Appendix B for comparative performance visualization across models).

To further assess predictive reliability under deployment-like conditions, bootstrap-based 95% confidence intervals were computed for each performance metric on the 2024 future holdout dataset. The resulting uncertainty estimates are summarized in Table 6.

As shown in Table 6, the confidence intervals for Quadratic Weighted Kappa, Macro-F1, Balanced Accuracy, and Ordinal MAE are not excessively wide, suggesting that model performance is not overly sensitive to random resampling and may be considered relatively stable. Importantly, the relatively narrow uncertainty bounds indicate that the observed performance under temporal validation is reproducible and not driven by sampling noise.

Taken together, these findings demonstrate that the proposed ordinal framework exhibits promising methodological properties, including stability of decision logic, temporal consistency of the explanatory structure, and controlled predictive uncertainty, all of which are essential prerequisites for clinical decision support. However, the raw predictive performance (QWK = 0.113, AUC = 0.512) remains insufficient for safe clinical deployment. While these reliability properties are necessary, they are not sufficient for real-world application, and further development is required before operational use can be considered.

## 5. Discussion

### 5.1. Temporal Generalization and Interpretation of Real-World Performance

The performance observed on the 2024 future holdout dataset was modest, presenting a markedly different picture from the high predictive metrics frequently reported in studies based on random data splitting. Rather than indicating model inadequacy, this result should be interpreted as a realistic estimate of performance under genuine deployment conditions, where temporal shifts, evolving disease patterns, and changes in clinical practice inevitably reduce predictive accuracy.

The 2024 data incorporate seasonal variation, shifts in disease composition, and temporal changes in clinical workflows, all of which introduce additional complexity when models are evaluated prospectively. This temporally grounded evaluation provides a more reliable estimate of real-world performance compared to conventional validation approaches, which are often prone to optimistic bias. From a public health perspective, such realistic estimates are valuable for understanding the limitations of current models. Before models can reliably support hospital capacity planning and resource allocation, predictive performance must be substantially improved through the inclusion of richer clinical data. To further contextualize model performance, we compared results against a naive baseline predictor (majority class assignment), which yielded substantially lower ordinal agreement, indicating that the proposed models capture non-trivial predictive structure beyond trivial classification. In addition, recent studies employing temporal validation in clinical prediction settings have similarly reported substantial performance degradation compared to random split designs, supporting the interpretation of modest performance under realistic conditions.

Despite the reduction in absolute performance levels, the relative ranking of models and the overall structure of prediction errors remained largely preserved. This consistency suggests that the models’ underlying decision logic is robust and not driven by random variation or overfitting. In clinical decision-support contexts, such stability is often more valuable than marginal improvements in accuracy, particularly in environments characterized by fluctuating infectious disease burden.

### 5.2. Ordinal Modeling, Error Structure, and Clinical Safety

The ordinal modeling framework provides a meaningful extension beyond binary or nominal classification by preserving the ordered nature of clinical recovery outcomes. Unlike binary models, which collapse heterogeneous recovery trajectories into a single category, ordinal models retain clinically relevant gradations of patient improvement.

The results demonstrate that most prediction errors occur between adjacent recovery levels, particularly between levels 1 and 2, while clinically critical extreme misclassifications are not observed. This pattern indicates that the model captures structured relationships in the data rather than producing random or arbitrary predictions.

Further analysis of the absolute error distribution shows that most predictions either match the true recovery level or deviate by only one ordinal category. Although overall performance metrics such as Macro-F1 and Balanced Accuracy are modest, the structured nature of prediction errors suggests that the model retains clinically meaningful information beyond what is captured by aggregate accuracy measures.

From a clinical risk perspective, misclassifications should be interpreted asymmetrically, where distant ordinal errors carry substantially higher clinical risk than adjacent-level deviations. Therefore, evaluating model performance solely based on overall accuracy may obscure clinically important differences in error severity.

From a safety perspective, the absence of large ordinal deviations indicates that the model avoids high-risk misclassification patterns that could lead to inappropriate clinical decisions. This property is particularly important in decision-support systems, where minimizing harmful errors is often more critical than maximizing overall accuracy.

In addition, the ordinal risk stratification framework has direct operational implications. Conceptually, stratifying patients according to predicted recovery levels could inform clinical attention and resource allocation. However, given the model’s modest discriminatory performance and the absence of key clinical variables, such stratification should be regarded as a theoretical framework rather than an operational recommendation. Prospective validation is required to assess whether predicted recovery levels translate meaningfully into clinical management decisions. From a conceptual standpoint, ordinal predictions structured around recovery levels could, in principle, map onto clinical triage categories used in admission-point decision-making. However, this framework has not been empirically tested in any clinical workflow, and the modest predictive performance observed (QWK = 0.113) indicates that predictions are not yet reliable enough for operational use. These ideas are presented as a conceptual framework and a direction for future applied research, not as a validated operational protocol.

### 5.3. Reliability, Stability of Explanations, and Trust

A key contribution of this study is the evaluation of model reliability beyond conventional performance metrics, incorporating both the stability of explanations and predictive uncertainty.

The bootstrap-based SHAP analysis demonstrates that the condition on admission (COA) consistently remains the most influential predictor across all resampled datasets. This finding is clinically intuitive and reinforces the alignment between model behavior and established medical reasoning. Seasonal and demographic variables also exhibit stable importance rankings, suggesting that the explanatory structure is not driven by noise.

The strong agreement between feature importance rankings in the training and holdout datasets further confirms that the model’s explanatory logic remains stable over time. Such temporal stability is essential for maintaining trust in clinical decision-support systems, particularly in dynamic healthcare environments.

Importantly, SHAP values quantify predictive contribution rather than statistical significance. Therefore, they should not be interpreted as substitutes for *p*-values or inferential statistics, but rather as complementary tools for understanding model behavior in predictive settings.

In addition, the relatively narrow confidence intervals for performance metrics indicate that the observed results reflect systematic patterns in the data rather than random variability. Together, these findings support the robustness, interpretability, and reliability of the proposed modeling framework.

To enhance clarity for clinical audiences, feature names in explanatory visualizations were revised to use full clinical terminology. This adjustment improves interpretability and facilitates practical use by non-technical healthcare professionals.

### 5.4. Limitations, Practical Implications, and Future Research Directions

This study has several important limitations that should be considered when interpreting the results.

First, the analysis is based on retrospective data from a single clinical center (the Third Department of the National Cancer Center, Mongolia). This is a primary limitation that substantially constrains external validity. Model performance, feature importance patterns, and calibration may differ substantially across hospitals with different patient populations, disease mixes, clinical protocols, and documentation practices. The findings cannot be generalized to other hospital types, regions, or healthcare systems without explicit multi-center validation. Second, the set of input variables is restricted to admission-time information (see Table A4 in Appendix C for detailed variable scope and missing features). Key clinical variables such as vital signs, laboratory results, comorbidities, inflammatory markers, and treatment data are not included. This limitation likely constrains predictive performance and may partially explain the modest accuracy observed.

Third, the outcome definition—based on the difference between COA and COD—provides an operationally convenient summary of patient improvement but does not capture intermediate clinical dynamics. This is a significant source of potential operational bias: the recorded COA and COD values may be influenced by institutional discharge practices, physician documentation habits, and clinical decision-making conventions at the single study center, rather than reflecting purely biological recovery. Readers should interpret recovery levels accordingly, recognizing that the ordinal outcome partially encodes institutional and procedural factors beyond patient physiology. Fourth, subgroup analysis indicates lower predictive performance in viral cases compared to bacterial cases. This heterogeneity may reflect the inherently higher variability and less predictable clinical progression of viral infections, as well as differences in diagnostic certainty and treatment standardization.

Fifth, the low proportion of patients in the highest recovery category (approximately 1.2%) introduces class imbalance (see Table A5 in Appendix D for detailed characteristics of the rare class). This may limit the model’s ability to detect rare but clinically important cases of exceptional recovery.

Finally, and critically, no empirical evaluation of the model’s impact on clinical workflows, bed management, or workforce allocation was conducted. All statements regarding capacity planning, treatment prioritization, and resource management represent conceptual directions and hypothetical applications rather than demonstrated outcomes. The model has not been integrated into any clinical workflow, and its operational impact remains untested. These applications should therefore be framed strictly as future research directions. As a retrospective study, the findings require prospective validation in real clinical workflows. Future research should focus on integrating richer clinical data, conducting multi-center studies, and linking predictive outputs to measurable operational indicators. Such efforts will be essential to fully realize the potential of ordinal machine learning models in practical healthcare settings.

## 6. Conclusions

This study defined improvement in the clinical course of acute infectious diseases as an ordinal outcome and developed a machine-learning framework for evaluation under conditions close to real-world clinical practice. A key strength of the study is the use of a strict temporal design, in which models trained on 2022–2023 data were evaluated on a fully isolated future dataset from 2024, thus enabling a realistic assessment of temporal generalizability. By grounding model evaluation in a temporally separated cohort, the study provides performance estimates that are more aligned with real-world health system conditions and public health preparedness needs.

The results indicate that ordinal modeling preserves the hierarchical nature of recovery outcomes and systematically avoids clinically high-risk extreme misclassifications. Although overall performance under the future holdout setting was modest, the error structure was largely concentrated between adjacent recovery levels, reflecting patterns that are clinically interpretable and acceptable. In addition, the study placed particular emphasis on model trustworthiness by evaluating the stability of feature importance, the temporal consistency of explanatory structures, and performance uncertainty. These analyses suggest that the models’ underlying decision logic remains largely stable across temporal shifts and that the observed performance reflects genuine data characteristics rather than random variability.

Importantly, model performance should be interpreted in the context of strict temporal validation and compared against trivial prediction strategies, as the proposed framework demonstrates non-trivial predictive structure beyond baseline classification despite modest aggregate metrics.

Such stability is a positive methodological indicator; however, achieving reliable risk stratification for hospital-level planning requires improved predictive accuracy and validation across multiple sites before operational deployment. This study demonstrates, at a proof-of-concept level, how ordinal predictions could conceptually be mapped to risk categories aligned with hospital operational logic. However, these mappings were not empirically tested against clinical workflows or operational outcomes. All such applications remain hypothetical and should be interpreted strictly as directions for future research rather than demonstrated capabilities of the current model. In practical terms, such incorporation into clinical workflows represents a future aspiration rather than a current capability. The modest predictive performance and the single-center, retrospective design of this study preclude any claims of clinical readiness. These operational applications require prospective validation, multi-center replication, and substantially richer predictor sets before they can be meaningfully evaluated. In addition, the outcome definition based on admission–discharge differences may incorporate elements of clinical decision-making and institutional practice and therefore should not be interpreted as a purely biological measure of recovery.

From a broader public health perspective, temporally validated ordinal modeling may strengthen hospital preparedness and system resilience during recurrent infectious disease waves. Nevertheless, further validation across multiple clinical settings and prospective evaluation within real-world workflows are required to confirm the generalizability and practical impact of the proposed approach.

Overall, this study should be regarded as a methodological proof-of-concept, demonstrating how ordinal clinical outcomes can be modeled in a time-aware and interpretable manner under strict temporal validation. Given the modest predictive performance (QWK < 0.2) and the exclusion of key clinical variables such as vital signs and laboratory results, the current framework is not yet suitable for direct clinical deployment. Future work incorporating richer clinical data, multi-center designs, and prospective evaluation is required before this approach could be considered for operational use.

## Figures and Tables

**Table 1:** Ordinal performance on the 2024 holdout dataset.

Model	QWK	Macro-F1	Balanced Accuracy	Ordinal MAE
Multinomial Logit	0.155	0.407	0.471	0.560
LightGBM	0.138	0.403	0.412	0.411
CatBoost	0.110	0.398	0.428	0.439
Gradient Boosting	0.102	0.414	0.467	0.447
Random Forest	0.090	0.398	0.400	0.511

Note: QWK = Quadratic Weighted Kappa; Macro-F1 = Macro-averaged F1 score; Balanced Accuracy = average recall across classes; Ordinal MAE = mean absolute error for ordinal predictions.

**Table 2:** Ordinal predictive performance stratified by disease etiology on the 2024 dataset.

Etiology	n	QWK	Macro-F1	Balanced Accuracy	Ordinal MAE
Viral	710	0.059	0.322	0.387	0.394
Bacterial	609	0.176	0.414	0.395	0.404
Other	293	0.185	0.449	0.514	0.468

Note: QWK = Quadratic Weighted Kappa; Macro-F1 = Macro-averaged F1 score; Balanced Accuracy = average recall across classes; Ordinal MAE = mean absolute error for ordinal predictions.

**Table 3:** Frequency of clinically high-risk misclassifications on the 2024 dataset.

Error Type	Count	Share of Total 2024	Share Within True = 3
Severe error: 3 → 0	0	0.0	0.00
Severe error: 3 → 1	0	0.0	0.00
Total severe errors: 3 → 1	0	0.0	0.00
Moderate-risk error: 3 → 2	3	0.19	15.79

**Table 4:** Comparison between ordinal (LightGBM) and binary modeling approaches on the 2024 holdout dataset.

Model	Metric	Value
Ordinal (4-level)-LightGBM	QWK	0.113
Binary (Improved vs. Not)	AUC	0.512

**Table 5:** Stability of feature importance rankings based on bootstrap SHAP analysis.

Feature	Mean |SHAP|	Mean Rank	Rank SD	Top-10 Frequency (%)
Condition on Admission	1.24166	1.00	0.00	100.0
Month of Admission	0.52409	2.42	0.49	100.0
Patient Age	0.51770	2.58	0.49	100.0
District of Residence	0.31141	4.02	0.13	100.0
Sex	0.19007	5.45	0.53	100.0
Insurance Status	0.17969	5.53	0.50	100.0
Ulaanbaatar Residency Status	0.02757	7.48	0.74	100.0
Payment Method	0.01851	8.07	0.65	100.0
Pregnancy Status	0.01150	8.90	1.14	100.0
Surgical Intervention (at admission)	0.00000	10.28	0.25	45.0

Note: SHAP values represent the average contribution of each feature to model predictions across bootstrap samples.

**Table 6:** Bootstrap 95% confidence intervals for the LightGBM ordinal model on the 2024 holdout dataset.

Metric	Estimate (2024)	95% CI (Lower)	95% CI (Upper)
QWK	0.111	0.061	0.160
Macro-F1	0.393	0.339	0.435
Balanced Accuracy	0.403	0.342	0.457
Ordinal MAE	0.462	0.438	0.489

## Data Availability

The clinical data used in this study are not publicly available due to privacy and ethical restrictions. The analytical workflow, feature engineering logic, and evaluation protocols are available from the author upon reasonable request.

## Appendix A. Outcome Structure and Data Distribution

This appendix provides additional descriptive statistics on the outcome variable and disease composition, supporting the interpretation of ordinal modeling results.

**Table A1:** Distribution of recovery levels in the 2024 holdout dataset (n = 1612).

Recovery Level	Count	Percentage (%)
0	10	0.6
1	569	35.3
2	1014	62.9
3	19	1.2

**Table A2:** Distribution of disease etiology.

Etiology	Count	Percentage (%)
Viral	3354	66.2
Bacterial	1299	25.6
Other	413	8.2

**Table A3:** Dataset split for temporal validation.

Dataset	Observations
Training set (2022–2023)	3454
Test set (2024)	1612

## Appendix B. Additional Feature Importance Analysis

This appendix presents supplementary feature importance analyses using alternative methods to complement the SHAP-based interpretation presented in the main text.

## Appendix C. Variable Scope and Limitations

This appendix summarizes the scope of variables included in the study and highlights key limitations related to omitted clinical features.

**Table A4:** Included and missing variables.

Category	Included Variables	Missing Variables
Demographic	age, sex	BMI, lifestyle factors
Clinical status	condition on admission (COA)	vital signs (BP, oxygen saturation)
Laboratory	-	CRP, WBC, biomarkers
Treatment	-	medication, antibiotics
Socioeconomic	insurance, payment status	income, education

The absence of detailed clinical and laboratory variables may limit predictive performance and partially explain the modest model accuracy observed in the main analysis. However, the use of admission-time variables ensures practical applicability under real-world deployment constraints.

## Appendix D. Rare Class Analysis

This appendix provides additional information on the low-frequency recovery level (level 3), which may influence model performance.

**Table A5:** Characteristics of recovery level 3 cases.

Metric	Value
Count	74
Percentage	1.2%
Mean age	36.1
Mean COA	2.04

The low proportion of patients in recovery level 3 introduces class imbalance, which may affect model sensitivity for extreme outcomes. This limitation is consistent with the observed performance patterns and is further discussed in the main text.
